# Optimisation of Fixed-Outlet and Flow-Modulated Pressure Reduction Measures in Looped Water Distribution Networks Constrained by Fire-Fighting Capacity Requirements

**DOI:** 10.3390/ijerph18137088

**Published:** 2021-07-02

**Authors:** Marius Møller Rokstad

**Affiliations:** Department of Civil and Environmental Engineering, Norwegian University of Science and Technology, S.P. Andersens veg 5, 7031 Trondheim, Norway; marius.rokstad@ntnu.no; Tel.: +47-953-30-206

**Keywords:** pressure management, leakage reduction, multi-objective optimisation, drinking water distribution modelling

## Abstract

Pressure management is a pivotal component when reducing leakages from water distribution networks, and can be achieved by sub-dividing existing networks into partitions where the pressure can be reduced effectively. There is a need to develop methods that aid in the identification of cost-effective partitions for pressure reduction, while simultaneously verifying that the topological changes entailed in these solutions do not compromise reliability and (fire-fighting) capacity requirements, especially in systems where the capacity is ensured through looped networks. This paper presents a method that can be used to this end, in which a novel combination of hydraulic simulations and graph theory is used to determine the maximal potential for (dynamic and static) pressure reduction, and this is used as a constraint for multi-objective optimization of pressure reduction measures. Trondheim, Norway, has been used as a case study area, and it is demonstrated how the developed method aids in the process of achieving leakage reduction in Trondheim. The results for Trondheim show that an economically optimal solution for pressure management is predicted to lead to a reduction from 28 to 22% water loss volume, and furthermore that effective pressure management will rely heavily on active (dynamic) regulation in this particular system.

## 1. Introduction

Norway is a country experiencing a low level of water stress [1], with easy access to suitable drinking water sources. The current proportion of drinking water loss volume in Norway is 29.8% [2], where continuous (background) leakage is the main contributor. The water loss in Norwegian water distribution networks (WDNs) is not extreme in a global context [3], but nevertheless considerably higher than its neighbouring Northern European countries [4]. Although a nation with low water stress can accept a higher level of water loss, the public acceptance is decreasing in Norway [5]. Many WDN managers are therefore working on reducing their water losses, and the four largest municipalities in Norway all have an explicitly stated strategic goal of reducing their leakage loss to 20% [6,7,8,9].

Pressure management is generally considered to be a highly cost-effective measure for reducing background leakage loss [10,11,12,13], since the leakage flow rate is dependent on some exponent of the pressure [14,15,16], and pressure reduction should as such be a viable measure for Norwegian WDNs to ascertain their leakage reduction goals. Effective implementation of pressure management strategies in existing WDNs is contingent on the ability to effectively divide existing pressure zones into smaller pressure management areas (PMAs) in which there is a high potential for implementing pressure reduction measures. Numerous methods for optimising the subdivision of WDNs have been developed (see, e.g., [17,18,19,20,21,22,23,24,25,26,27,28]), for example, by minimising the number of cuts and maximising, e.g., pressure uniformity within each district metering area (DMA). These methods could also be used to optimise the configuration of PMAs in Norwegian WDNs. However, even though there is great potential for applying pressure management to achieve leakage reduction in Norwegian WDNs, this opportunity has not yet been explored systematically by any of the larger WDN managers, mainly because of the following characteristics specific for Norwegian WDN operation [29]:

- Norwegian WDNs have high requirements for fire-fighting capacities, with little degree of distinction between different area types. The general requirements in the current regulations [30] state that the fire-fighting capacity should be 20 L/s for individual housing areas, and 50 L/s for other areas. Earlier legislation only required 12 L/s for individual housing areas. In practice, the current regulations imply that the capacity is 50 L/s for all areas which are not solely consisting of individual housings. High-density and industrial areas may have higher requirements, depending on the risk evaluation of the local fire department. The fire-fighting capacity requirements come in addition to the design water consumption, which implies that many pipes in Norwegian WDNs are (grossly) over-dimensioned with respect to normal demand situations.
- The WDNs often depend on a high degree of loopedness to satisfy said fire-fighting requirements, i.e., that the required capacity in certain nodes is satisfied by multiple flow paths (loops) between source(s) and demand points. Typically, the utilisation of multiple flow paths may not be necessary to maintain minimal service pressures under normal demand situations, but only be necessary in the event that there is a fire-flow extraction from the network.
- Norway is hilly, and many existing pressure zones cover large elevation spans, which means that the hydraulic capacity in one point may be limited by pressure-deficient conditions at another point (e.g., with higher elevation) in the system.

Thus, if one is going to optimise the delineation of PMAs within an existing complex WDN, one has to ensure that the explicitly defined fire-fighting capacity is not compromised in any of the nodes in the WDN due to the topological changes (e.g., installation of valves and closing of pipes) caused by the subdivision of the network. Since the currently available methods for DMA optimisation do not take these fire-fighting capacity requirements into account, one cannot simply use any of these methods to identify PMAs in Norwegian WDNs, as the application of these methods may lead to solutions which may violate the fire-fighting capacity requirement in one or more nodes. Because the WDNs typically are over-dimensioned with respect to normal demand, and depend on looped structures only in the occurrence of fire-fighting demands, one cannot in general predict if a certain solution for partitioning the network will result in compromising fire-fighting capacities without performing hydraulic simulations. Since the many of the developed methods rely on evaluating numerous candidate solutions, and hydraulic simulations must be performed for each node with a defined capacity requirement for each candidate solution, it becomes computationally cumbersome to include this constraint in a network partitioning optimisation algorithm. For illustration, if one has a WDN where fire-fighting capacity needs to be maintained in 10,000 nodes, and one wants to optimise the partition of this WDN using, e.g., a genetic algorithm with 1000 candidate solutions over 500 generations, the number of hydraulic simulations needed to verify that the nodal demands are met will be 10,000 × 1000 × 500 = 5 $\times \;10^{9}$; if each simulation takes 1 s, this amounts to 158 years of computation time.

At present, there are numerous solutions available for modelling WDNs hydraulics to determine minimum pressures to sustain adequate flow demands for fire-fighting (see, e.g., [31,32,33]). Likewise, there exists numerous examples of successful applications of WDN partitioning optimisation algorithms aimed at achieving leakage loss reduction (see, e.g., [17,18,19,20,21,22,23,24,25,26,27,28]). However, because of the aforementioned reasons, there exists no solution that combines these two parts, i.e., optimisation of PMA partitioning combined with hydraulic simulation to verify that fire-fighting capacities are maintained due to the topological changes imposed by the introduction of the PMAs. Current decision support systems therefore rely on labour-intensive simulation modelling for identification of effective ways of dividing WDNs with the purpose of pressure reduction and to ensure that the fire-fighting capacity is not compromised. Norwegian WDN managers are therefore generally reluctant to implement any pressure management schemes that involve any topological changes in their networks. Having already identified the areas that are obviously beneficial to isolate for pressure reduction purposes, the utility managers are unaware how to best proceed with the remaining sub-systems of their WDNs: they do not know where in these looped sub-systems it would most efficient to delineate new PMAs, and given an arbitrary configuration of PMAs it is not obvious which nodes would be considered critical nodes; verifying that the fire-fighting capacity is maintained in all nodes therefore requires a high number of hydraulic simulations for any candidate partitioning solution. In order to enable the identification and implementation of effective and feasible network partitioning solutions, there is a need to develop a method that takes into account the above-mentioned constraints.

Trondheim, Norway’s 3rd largest city, with approximately 200,000 inhabitants [8] and an average population density of 3250 pop./km^2^ [34], has been working systematically on reducing the leakage loss from the municipal WDN for the past three decades. The average demand in Trondheim’s WDN is around 720 L/s (including losses), which is distributed through 814.47 km of municipal pipe network, covering approximately 65 pressure zones. The leakage loss in Trondheim was approximately 45–55% by the end of the 1980’s, but the leakage proportion has been reduced to 28% in the period 1990–2015, by:

- Installing DMAs for quick detection and repair of bursts;
- Employing systematic active leakage detection programs;
- Targeted renewal of leaky pipes;
- A general increase in buried infrastructure renewal, e.g., in combination with the City’s road resurfacing program.

Trondheim has a strategic goal of reducing the leakage level further down to 20% by 2028 [8], in line with the national strategy [5]. However, despite continuing the aforementioned efforts, Trondheim has for the past five years experienced a stagnation in the leakage reduction. Although the leakage could further be reduced by intensifying one or more of the above mentioned strategies, it is highly likely that Trondheim must also include systematic pressure management strategies in order to achieve the leakage level goal in a cost-effective manner, especially considering that the current average pressure (68 mH_2_O (667 kPa)) is much higher than the minimally required service pressure (30 mH_2_O (294 kPa)) in the system. However, pressure management has not yet been systematically applied in Trondheim, since the City is reluctant to do labour-intensive simulation modelling to further sectorize the WDN for optimizing the pressure zones, which may require capital expense with additional booster stations to ensure that the fire-fighting capacities are not violated. This paper summarises a case study in which such a novel method has been developed and applied to partition the Trondheim WDN with the purpose of optimising the system’s pressure management. Some key properties of the developed method are:

- Partitioning of new PMAs is performed within existing pressure zones of the WDN; the current structure of the system is in other words maintained;
- Hydraulic grade lines necessary for maintaining the system’s function (capacity) requirements are conserved. Fire-fighting capacity requirements are therefore maintained and the resulting solutions do not infer the need to install new additional pumps in the WDN;
- Flow-modulated (FM) pressure regulation may be a viable, cost-effective approach for certain partitions of networks which have high fire-fighting requirements [12], as is the case for the Trondheim WDN, while traditional fixed outlet (FO) or static pressure reduction may be the most effective for other areas. The developed method therefore also optimises the allocation of either FO or FM pressure-regulation for the partitioned WDNs by distinguishing between costs and benefits of the two pressure-regulation approaches;
- The method aims at minimising the cost of installing valves to partition the network, while maximising the pressure reduction (and consequently the leakage reduction) made possible by the partitioning.

The developed method is based on novel approaches for handling the fire-fighting capacity constraint (namely graph theory in conjunction with hydraulic simulations) with well-known methods for optimising the partitioning of new PMAs (namely the non-dominated sorting algorithm (NSGA-II)), the product of which will be demonstrated in a case study presented in this paper.

## 2. Materials and Methods

### 2.1. Decision Objectives and Constraints

The overall objective of the developed method is to identify cost-effective ways of partitioning a network for the purpose of reducing the pressure, and consequently the leakage level, i.e.,

O1 maximising the reduction pressure-dependent component of the background leakage ($\Delta Q_{leak}\propto P^{\alpha }$), and
O2 minimising the cost (*c*) of the cuts (valve locations) that need to be installed in the system to achieve the partitioning solution.

The decision problem is constrained by:

- The current structure of the WDN should be maintained, i.e., solutions for partitioning the network should be contained within existing pressure zones, and hydraulic grade lines necessary for maintaining system service level should be preserved;
- The required fire-fighting capacities in each node should not be compromised.

The method should furthermore consider both FO/static and FM/dynamic pressure regulation as options for identified PMAs, since these two approaches are associated with different costs (of installation and maintenance) and benefits (achieved pressure reduction). It is believed that FM pressure regulation may be beneficial for some areas of the WDNs in question, whereas static control may be more suitable for others.

**Figure 1:**
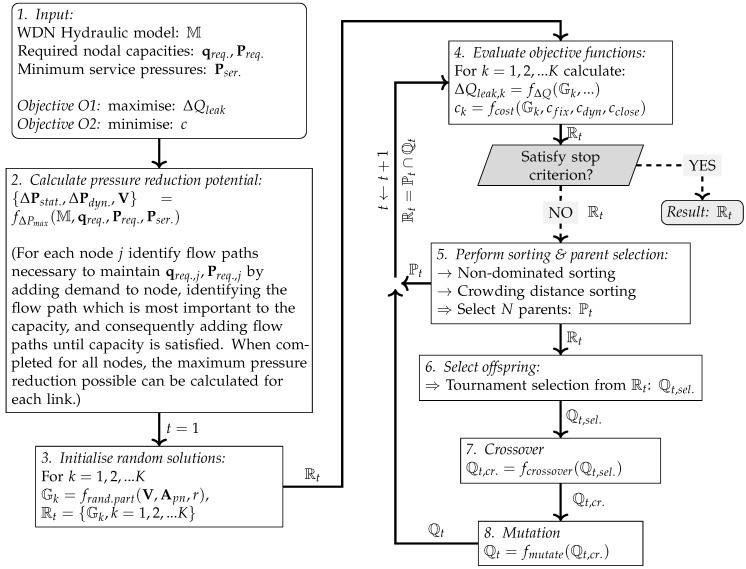
Flow chart with suggested algorithm.

To achieve this, the approach depicted in Figure 1 was adopted. First, the novel algorithm for determining the maximum pressure reduction potentials was applied (step 1–2 in Figure 1, explained in Section 2.2). Using the calculated pressure reduction potentials as constraints, the NSGA-II algorithm was used to optimise the partitioning of the network (step 3–7 in Figure 1, explained in Section 2.3). The approach in Figure 1 will be explained further in the following sections.

The algorithms described in this paper were implemented in MATLAB, using a EPANET 2.0 [31] wrapper to perform hydraulic calculations.

### 2.2. Constraints from Fire-Fighting Capacity Requirements

In order to handle the limitations on pressure reduction potential imposed from the fire-fighting capacity requirements, and simultaneously avoiding the need for performing hydraulic analyses to check for fire-fighting capacity for every candidate partitioning solution, an algorithm has been developed which identifies which links are necessary to preserve the required capacity in each node in the system. Based on the resulting dependencies different nodes inflict on each link, one can determine which links (and in which direction) one can install pressure reduction valves, and to what extent one can maximally reduce the pressure in each link, assuming either static or FM pressure regulation. The results of the algorithm thus allows one to treat the fire-fighting capacity requirements as a constraint in the optimisation, and eliminates the need to perform hydraulic simulations to verify that the required capacities are maintained for each evaluated candidate partitioning solution.

The idea of the method is to combine hydraulic simulations with graph theory to identify the minimal set of paths that are necessary to maintain the required capacity in any given node in the system. When the paths (set of links and nodes) identified as the minimum required to fulfil the hydraulic capacity requirements of a specific node, one knows that one cannot change the topology of the network so that these paths do not supply water to the specific node. Based on this, and repeating this exercise for all nodes, one can calculate where and how much one can maximally reduce the pressure of each network element.

**Figure A1:**
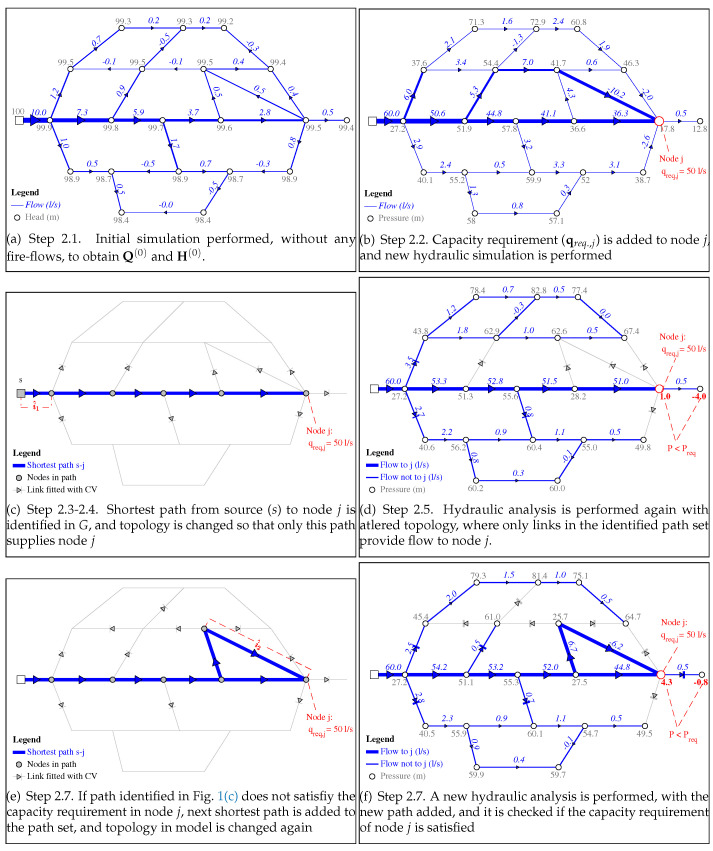
(**a**) Step 2.1. Initial simulation performed, without any fire-flows, to obtain $Q^{\left(0\right)}$ and $H^{\left(0\right)}$. (**b**) Step 2.2. Capacity requirement ($q_{req.,j}$) is added to node *j*, and new hydraulic simulation is performed. (**c**) Step 2.3–2.4. Shortest path from source (*s*) to node *j* is identified in *G*, and topology is changed so that only this path supplies node *j*. (**d**) Step 2.5. Hydraulic analysis is performed again with altered topology, where only links in the identified path set provide flow to node *j*. (**e**) Step 2.6. If the path identified in Figure 1c does not satisfy the capacity requirement in node *j*, the next shortest path is added to the path set, and the topology in model is changed again. (**f**) Step 2.7. A new hydraulic analysis is performed, with the new path added, and it is checked if the capacity requirement of node *j* is satisfied.

Since the algorithm to achieve this has already been described in [29], and due to space limitations, this method will not be described in detail in this paper. However, the method is described in short below, and it is referred to Figure A1 (Appendix A) for a visual illustration of how the algorithm works:

1 The hydraulic model of the network, M, is represented as a graph *G*, in which each link is represented as an edge, and each node as a vertex. The network model is associated with a set of capacity requirements, defined in $q_{req.}$, $P_{req.}$, and $P_{ser.}$. $q_{req.,j}$ is the (fire) flow capacity requirement in node *j*, which needs to be delivered at a minimal pressure of $P_{req.,j}$, while $P_{ser.,j}$ is the minimum acceptable service pressure during normal (non-firefighting) operation.
2.1 An initial hydraulic simulation is run, to assess steady state flows and heads, $Q^{\left(0\right)}$ and $H^{\left(0\right)}$, respectively, without any fire-fighting demand. (Figure A1a.)
2.2 For each node *j*, the capacity requirement $q_{req.,j}$ is added as a nodal demand, and a hydraulic simulation is run again, thus obtaining the flows and heads with the fire-flow demand, $Q^{\left(0,j\right)}$ and $H^{\left(0,j\right)}$. (Figure A1b.)
2.3 The edge weights $w_{i}$ in the graph *G* are updated, so that $w_{i}={\left|Q_{i}^{\left(0,j\right)}-Q_{i}^{\left(0\right)}\right|}^{-1},\forall i$. The edges that are influenced the most by the activation of the fire-fighting demand in node *j* (i.e. experiencing the largest absolute change of flow) are thus assigned the highest edge weights. The shortest path in *G*, with updated weights $w_{i}$, from any source, Z to node *j* is identified, i.e., the path which is most influential for the capacity in node *j*. The links in the shortest path are assigned to the set $L^{\left(j\right)}$ (the current path set). (Figure A1c.)
2.4 The topology of the WDN network model is changed (by using check valves) so that only the links $L^{\left(j\right)}$ can provide capacity to node *j*. (Figure A1c.)
2.5 The hydraulic model is run again with the changed topology to retain the flows and heads, $Q^{\left(1,j\right)}$ and $H^{\left(1,j\right)}$. (Figure A1d.)
2.6 If the capacity in node *j* is satisfied ($H_{k}^{\left(1,j\right)}\geq P_{req.,k}+z_{k},\forall k$) using only the links in the current path set, $L^{\left(j\right)}$ (i.e., the links highlighted in Figure A1c), the algorithm considers the links in the set $L^{\left(j\right)}$ as sufficient for providing the capacity in node *j*, and moves on to the next node in the system.
2.7 If the capacity in node *j* is not satisfied using only the links in the current path set $L^{\left(j\right)}$, the algorithm returns to step 2.3. The shortest path in *G* which is not member of $L^{\left(j\right)}$ is then added to $L^{\left(j\right)}$ (see Figure A1e), and a new hydraulic simulation is performed to check if the capacity requirement is satisfied (see Figure A1f). This process of adding paths contributing to the flow to node *j*, and calculating if the capacity requirement is met, is repeated until the capacity in node *j* is satisfied.

The above-outlined method allows one to identify which nodes depend on which links to maintain required fire-fighting capacity demands. The dependencies defined in $L^{\left(j\right)}$ and the flow directions in $Q^{\left(1,j\right)}$ represent constraints as to where and which direction one can install pressure reduction valves (PRVs) in the system. If a node depends on a certain link in order to maintain its capacity requirement, then that link cannot be assigned to a pressure zone with a hydraulic grade-line that is lower than the service pressure of the node, i.e., a link cannot be assigned to a lower pressure zone than the nodes by which it is required. By identifying these dependencies, one can calculate the maximum pressure reduction possible for each link in the system before optimising the partitioning of the network, and there will be no need to perform hydraulic simulations during the optimisation step, thus reducing the computational burden of the decision problem.

The algorithm yields the following output for each link *i* in the WDN:

- $\Delta P_{stat.,i}$: the maximum possible static pressure reduction in link *i*, i.e., reduction if FO pressure regulation is assumed;
- $\Delta P_{dyn.,i}$: the maximum possible dynamic pressure reduction in link *i*, i.e., assuming that, e.g., FM regulation is used (to reduce closing degree of valves in case of fire-fighting demand);
- $V_{i,1}=\left\{\begin{array}{cc} 1 & \mathrm{if}\;a\;\mathrm{valve}\;\mathrm{from}\;\mathrm{the}\;1\text{st}\;\mathrm{to}\;2\text{nd}\;\mathrm{node}\;\mathrm{of}\;\mathrm{link}\;i\;\mathrm{does}\;\mathrm{not}\;\mathrm{violate}\;\mathrm{any}\;\mathrm{capacity}\\ & \mathrm{requirement}\\ 0 & \mathrm{otherwise} \end{array}\right.\;V_{i,2}=\left\{\begin{array}{cc} 1 & \mathrm{if}\;a\;\mathrm{valve}\;\mathrm{from}\;\mathrm{the}\;2\text{nd}\;\mathrm{to}\;1\text{st}\;\mathrm{node}\;\mathrm{of}\;\mathrm{link}\;i\;\mathrm{does}\;\mathrm{not}\;\mathrm{violate}\;\mathrm{any}\;\mathrm{capacity}\\ & \mathrm{requirement}\\ 0 & \mathrm{otherwise}\;. \end{array}\right.$

See [29] for further details on how these parameters are calculated. For shorthand, the calling of the algorithm to determine the pressure reduction potentials is denoted $\left\{\Delta P_{stat.},\Delta P_{dyn.},V\right\}=f_{\Delta P_{max}}\left(M,q_{req.},P_{req.},P_{ser.}\right)$ in the flow chart in Figure 1.

To ensure that elevated storage tanks maintain their intended performance, i.e., that there is a sufficient capacity to fill the storage tanks with their respective design flows, storage tanks have been handled in a similar manner as for other nodes with capacity requirements in the algorithm. For each storage tank *j*, the design flow into the tank has been specified in $q_{req.,j}$, and the tank has been replaced by a junction node with demand $q_{req.,j}$ and a pressure requirement ($P_{req.,j}$) corresponding to the maximum water level of the tank. By applying the same above-mentioned algorithm as for the nodes with fire-fighting capacity requirement for the storage tanks, one ensures that their hydraulic function is maintained.

In the above-described algorithm, it is implicitly assumed that one maintains system function for all nodes in the WDN by ensuring that each node is served by the flow paths necessary to maintain fire-fighting capacity. However, not all nodes have a non-zero fire-fighting capacity requirement. In order for a partitioning solution to be valid, one must also ensure algebraic connectivity of junctions that do not have any (non-zero) fire-fighting capacity requirement associated with them, i.e., one must ensure that the junction is supplied by a source (either a reservoir or tank) after the partitioning solution has been implemented. In order to ensure this algebraic connectivity, a constraint is imposed that no junction should be cut off from the source which is hydraulically closest to it. Thus, the pressure in a link that is in the shortest supply path to a node cannot be reduced beyond the service pressure of any of the nodes it supplies. To calculate the shortest paths and identify the links on which the different nodes depend, a bi-directional weighted graph representation of the WDN is made, and the shortest path from any source to each junction is identified using Dijkstra’s algorithm [35]. The algorithm for this is outlined in Appendix A.2.

### 2.3. Optimising Configuration of New PMAs with NSGA-II

The optimisation algorithm initialises by generating a set of *K* different partitioning solutions at random, see step 3 in Figure 1. The random partitioning solutions are generated by seeding new partitions at random elements (links/nodes) in the network, identifying elements adjacent to the current partition (by means of the topological incidence matrix $A_{pn}$) which have not yet been assigned to another partition, and randomly choosing one of these to add to the current partitioning, and continuing adding elements to the current partition with probability *r*; when the probabilistic stopping criterion has been met, the algorithm starts a new partition at another unassigned element. Each partition is randomly assigned either as a statically or dynamically pressure controlled partition. The partitioning solutions are made so that cuts in the network are only made in locations and in directions that do not lead to a violation of the capacity requirements identified in the previous step, as defined in V. The generation of random partitioning solutions is denoted $G_{k}=f_{rand.part}\left(V,A_{pn},r\right)$ in Figure 1, where $G_{k}$ is a candidate solution for partitioning, defined in the following way:

(1) $$\begin{array}{cc} G_{k} & =\{S_{k},D_{k},C_{k}\}\\ S_{k} & =\{S_{k,1},S_{k,2},...,S_{k,j},...,S_{k,J_{S_{k}}}\}\\ D_{k} & =\{D_{k,1},D_{k,2},...,D_{k,j},...,D_{k,J_{D_{k}}}\} \end{array}$$

where $S_{k}$ refers to the set of PMAs which are to be controlled by static (FO) pressure regulation, and $D_{k}$ are to be controlled by dynamic (e.g., FM) pressure regulation. A link *i* can be member of maximally one set in $G_{k}$, thus:

- $S_{k,m}\cup S_{k,l}=\emptyset ,\forall m\neq l$,
- $D_{k,m}\cup D_{k,l}=\emptyset ,\forall m\neq l$, and
- $D_{k,m}\cup S_{k,l}=\emptyset ,\forall \left\{m,l\right\}$

The maximal pressure reduction possible for every $S_{k,j}$ defined in $S_{k}$ will be:

(2) $$\Delta P_{S_{k,j}}=\min\left[\Delta P_{stat.,i},\forall i\in S_{k,j}\right]$$

since the pressure cannot be reduced beyond the maximal potential of the link with the minimal potential for pressure reduction, without violating the required service pressure requirements for this link. Similarly, the maximal pressure reduction possible for $D_{k,j}$ will be:

(3) $$\Delta P_{D_{k,j}}=\min\left[\Delta P_{dyn.,i},\forall i\in D_{k,j}\right]$$

The maximum estimated leakage reduction made possible by a given partitioning solution defined in $G_{k}=\left\{S_{k},D_{k}\right\}$, i.e., the first objective of the decision problem, can thus be calculated as:

(4) $$\begin{array}{cc} \Delta Q_{leak,k}= & \sum_{j=1}^{J_{S_{k}}}\left[\sum_{\forall i\in S_{k,j}}L_{i}\beta_{i}\left(P_{i}^{\alpha_{i}}-{\left(P_{i}-\Delta P_{S_{k,j}}\right)}^{\alpha_{i}}\right)\right]+\sum_{j=1}^{J_{D_{k}}}\left[\sum_{\forall i\in D_{k,j}}L_{i}\beta_{i}\left(P_{i}^{\alpha_{i}}-{\left(P_{i}-P_{D_{k,j}}\right)}^{\alpha_{i}}\right)\right] \end{array}$$

The calculation of $\Delta Q_{leak,k}$, as outlined in Equations (2)–(4) is denoted as $\Delta Q_{leak,k}=f_{\Delta Q}\left(G_{k},\alpha ,\beta ,P,L,\Delta P_{stat.},\Delta P_{dyn.}\right)=f_{\Delta Q}\left(G_{k},...\right)$.

The $\left[K\times n_{p}\times 2\right]$ matrix C defines the configuration of cuts between the partitions in $G_{k},\forall k$:

$$\begin{array}{cc} C_{k,i,1} & =\left\{\begin{array}{cc} 1 & \mathrm{if}\;\mathrm{there}\;\mathrm{is}\;a\;\mathrm{valve}\;\mathrm{by}\;\mathrm{the}\;1\text{st}\;\mathrm{node}\;\mathrm{of}\;\mathrm{link}\;i\;\mathrm{in}\;\mathrm{direction}\;\mathrm{from}\;1\text{st}\;\mathrm{to}\;2\text{nd}\\ & \mathrm{node}\;\mathrm{of}\;\mathrm{link}\\ -1 & \mathrm{if}\;\mathrm{there}\;\mathrm{is}\;a\;\mathrm{valve}\;\mathrm{by}\;\mathrm{the}\;1\text{st}\;\mathrm{node}\;\mathrm{of}\;\mathrm{link}\;i\;\mathrm{in}\;\mathrm{direction}\;\mathrm{from}\;2\text{nd}\;\mathrm{to}\;1\text{nd}\\ & \;\mathrm{node}\;\mathrm{of}\;\mathrm{link}\\ 0 & \mathrm{otherwise} \end{array}\right.\\ C_{k,i,2} & =\left\{\begin{array}{cc} 1 & \mathrm{if}\;\mathrm{there}\;\mathrm{is}\;a\;\mathrm{valve}\;\mathrm{by}\;\mathrm{the}\;2\text{nd}\;\mathrm{node}\;\mathrm{of}\;\mathrm{link}\;i\;\mathrm{in}\;\mathrm{direction}\;\mathrm{from}\;1\text{st}\;\mathrm{to}\;2\text{nd}\\ & \;\mathrm{node}\;\mathrm{of}\;\mathrm{link}\\ -1 & \mathrm{if}\;\mathrm{there}\;\mathrm{is}\;a\;\mathrm{valve}\;\mathrm{by}\;\mathrm{the}\;2\text{nd}\;\mathrm{node}\;\mathrm{of}\;\mathrm{link}\;i\;\mathrm{in}\;\mathrm{direction}\;\mathrm{from}\;2\text{nd}\;\mathrm{to}\;1\text{nd}\\ & \;\mathrm{node}\;\mathrm{of}\;\mathrm{link}\\ 0 & \mathrm{otherwise} \end{array}\right. \end{array}$$

The values in $C_{k,:,:}$ can be used to calculate the cost of the valves that need to be installed in order to achieve the partitions defined in $G_{k}$. The function $c_{k}=f_{cost}\left(G_{k},c_{fix},c_{dyn.},c_{closed}\right)$ calculates the cost of the valves that need to be installed in order to achieve the partitioning in $G_{k}$. The function goes through all non-zero instances in $C_{k,:,:}$, and determines the function of each valve defined in $C_{k,:,:}$:

- If a valve in $C_{k,:,:}$ feeds into a partition that is defined to by dynamically controlled in $G_{k}$, a FM PRV is chosen, and the cost $c_{dyn}$ is added to $c_{k}$;
- If a valve $C_{k,:,:}$ feeds into a partition that is defined to by statically controlled in $G_{k}$, a FO PRV is chosen, and the cost $c_{fix}$ is added to $c_{k}$;
- If a valve is $C_{k,:,:}$ is not necessary to maintain the required capacity in any node (i.e., $V_{k,1}=V_{k,2}=1$), the pipe can be closed, and the cost of installing an isolation valve $c_{close}$ is added to $c_{k}$.

For example, a partitioning solution $G_{k}$ that requires 4 PRVs feeding into dynamically controlled PMAs, 3 PRVs feeding into statically controlled PMAs, and 2 closed isolation valves will have the cost $c_{k}=4\cdot c_{dyn.}+3\cdot c_{fix.}+2\cdot c_{close}$.

The remaining part of the algorithm depicted in Figure 1 (step 5–8.) follows a fairly standard NSGA-II scheme [36], where:

5. The candidate solutions are sorted according to non-dominance and crowding distance, and the top *N* are chosen as parents ($P_{t}$) for the next generation;
6. Offspring are propagated by tournament selection from the list of ordered solutions $R_{t}$, for
7. Crossover and
8. Mutation.

Selected, crossovered and mutated solutions are stored in the sets $Q_{t,sel}$, $Q_{t,cr.}$ and $Q_{t}$, respectively. After this, the algorithm moves to the next generation, evaluates the objective functions of the new candidate solutions (step 4), before repeating the process unless the stop criterion has been satisfied.

## 3. Results

### 3.1. Input Data from Case Study Area

The hydraulic model representation of the case study area WDN, which consisted of $n_{n}=$ 8809 nodes $n_{p}=$ 10,018 links, with associated fire-fighting capacity requirements and required service pressures, was used as input for the analysis. The input model contained all currently existing PRVs and closed pipes of the existing system; the currently existing DMAs and PMAs of the WDN were thus considered in the analysis. The fire-fighting capacity requirements in Trondheim are aligned with the national requirements, and were consequently set to either 0, 12, 20 or 50 L/s, depending on the type of area around each particular node (although the current requirements are either 20 or 50 L/s, there are certain areas in Trondheim which have been approved for development under older legislation, where the requirement was only 12 L/s). The minimal required service pressure for the system is 30 mH_2_O (294 kPa) under normal diurnal demand variation, and minimally 10 mH_2_O (98 kPa) in fire-fighting scenarios. The minimum pressure requirements are the same regardless of the time of day.

The WDN provided by the Trondheim municipality has been calibrated with respect to demand and leakage loss, where the leakage loss has been estimated based on readings from each DMA, using the International Water Association’s standard water balance method [37]. The estimate of 28% water loss includes real and apparent losses. The effect of pressure reduction measures, in terms of reduced leakage flow, was predicted and evaluated using Equation (4), based on the WDN’s calibrated leakage parameters [α,β].

To keep the presentations of the results conceptually simple, the costs of the valves have been set as: $c_{fix}=1$, $c_{dyn}=2$, and $c_{close}=0$. Real costs for installation of valves have thus not been used, but by assigning the values 1 and 2 to $c_{fix}$ and $c_{dyn}$, respectively, it is assumed that the installation of a FO PRV has the cost of one cut, whereas the installation of an FM PRV is twice as costly. For easy comparability of the results, 1 FO PRV is henceforth referred to as 1 valve equivalent, while 2 FO PRV is referred to as 2 valve equivalents. By assigning $c_{close}=0$, one implicitly assumes that the there are isolation valves present at the points where one wants to close the system, and that the closing of these valves does not imply any investment cost.

When making practical decisions about whether to choose FO- or FM-based PRVs in a system, other factors are also evaluated, in addition to just investment cost and the magnitude of achieved pressure reduction. Typically, establishing FM PRVs is more challenging (compared to FO PRVs) when it comes to operability, inspection and maintenance needs, implementation of appropriate real-time control (RTC) strategies and Supervisory Control And Data Acquisition (SCADA) system integration. However, for simplicity of presentation of the results in this paper, only cost and pressure reduction benefits are considered.

### 3.2. Pressure Reduction Potential Results

**Figure 2:**
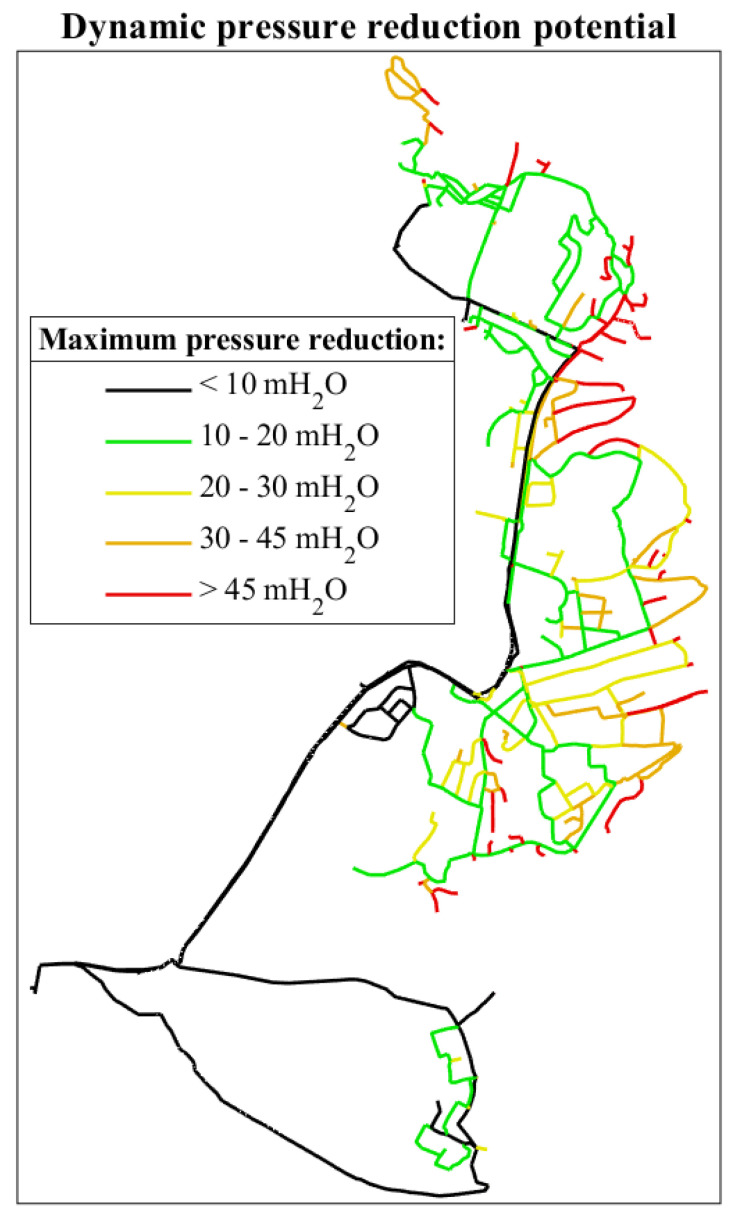
Maximal dynamic pressure reduction potential for a portion of Trondheim’s WDN, from [29].

The described method for calculating the maximal potential for pressure reduction in each link, without compromising the fire-fighting capacity requirements, has been applied and demonstrated for Trondheim’s WDN, and the results are summarised in [29]. Figure 2, showing the maximal dynamic pressure reduction possible for the links in a part of Trondheim’s WDN, has been included to illustrate the results from the algorithm. The calculated static and dynamic pressure reduction potentials were obtained and used as input for the optimisation algorithm.

### 3.3. Optimisation Results from Example Zone in Trondheim

To illustrate how the proposed method has been applied, and what outcomes it has yielded, the partitioning optimisation of one existing pressure zone in Trondheim has been used as an example, namely zone no. 106. This zone accounts for approximately 9% of the total pipe network length in Trondheim.

**Figure 3:**
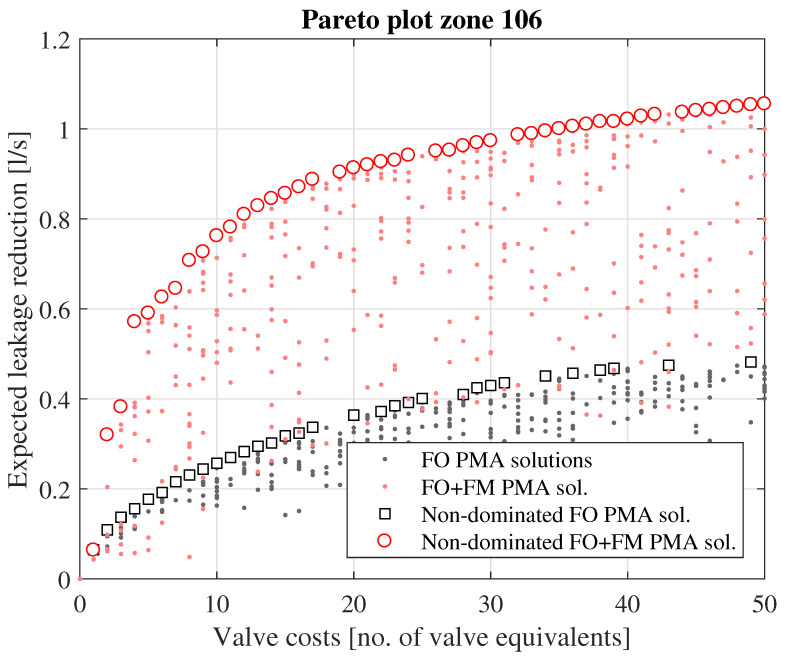
Resulting Pareto plot from example zone (no. 106) in Trondheim’s WDN.

The Pareto plot for zone no. 106 (Figure 3) demonstrates how pressure management could effectively be used to reduce the leakage loss in the zone, both for the case assuming only FO PRVs as well as the case assuming combinations of FO and FM PRVs. Generally, the results for this particular zone show that the application of FM PMAs will be more effective in terms of expected leakage reduction than FO PMAs. For instance, the optimal expected leakage reduction achieved when installing 4 FO PRVs is 0.158 L/s, as also shown in Table 1. For the same valve cost, i.e., installing 2 FM PRVs, the expected leakage reduction is 0.572 L/s, i.e., 360% more than what can be achieved using only FO PRVs. For FO only PMAs configuration, the maximal possible potential for leakage reduction is around 0.47 L/s (assuming > 50 PRVs installed), while the equivalent potential for a combination of FO and FM PMAs is around 1.15 L/s.

**Figure 4:**
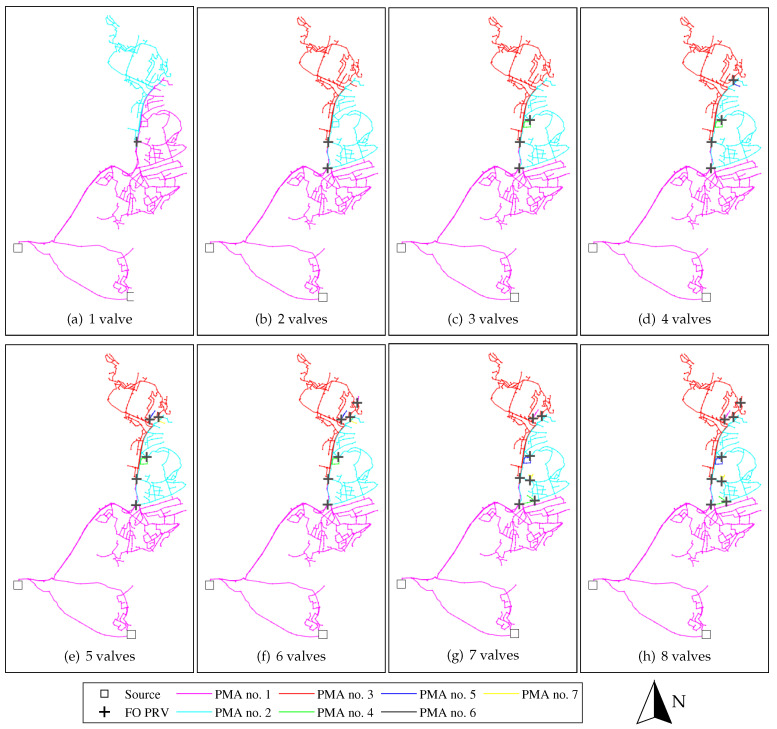
Optimal partitioning for 1–8 valves in example zone (no. 106), assuming only FO PRVs.

**Figure 5:**
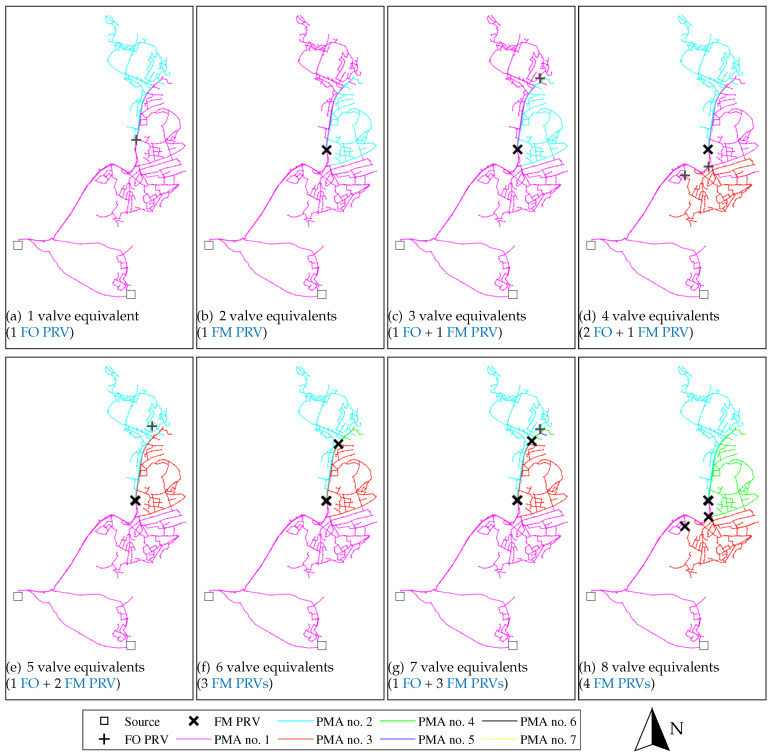
Optimal partitioning for 1–8 valve equivalents in example zone (no. 106), assuming a combination of FO and FM PRVs. (For Figure 5e and onwards, there are two FM PRVs placed right next to each other in the centre of the network, thus appearing as just one FM PRV.)

Figure 4 and Figure 5 show the identified Pareto optimal partitioning solutions for the first 1–8 valve costs (no. of valve equivalents), for FO only PMAs partitioning and for FO+FM PMAs, respectively. For the case considering only FO pressure reduction, the optimisation algorithm consistently identifies solutions that divide the existing zone into three large partitions; the first and second PRVs are used to partition the network into two and three partitions, respectively, each consisting of a combination looped and branched pipe structures. Dividing the system into these three partitions is thus considered the most effective utilisation of the two first FO PRVs, and this partition configuration is conserved in the Pareto optimal solutions with higher numbers of PRVs. For the solutions with more than two FO PRVs, the additional PRVs are used to create small, new PMAs primarily on branched pipe structures within the three PMAs identified as Pareto optimal for two FO PRVs. The fact that only very small, branched PMAs are identified beyond the three large partitions identified in the solution using 2 PRVs, indicates that there the potential for effective pressure management by means of FO PRVs is limited beyond these three partitions; it is likely that these additional small, branched PMAs are not cost-effective solutions.

The case with simultaneous optimisation of FO and FM PMAs (Figure 5) show comparable results to the FO PMA only case, although the partitioning and priorities of valve utilisation are not exactly the same. One may notice the similarity between the solution in Figure 4b (optimal solution with 2 FO PRVs) and Figure 5h (optimal solution with 8 FO + FM valve equivalents). The two PMAs identified in the northern end of the existing zone (cyan and green in Figure 5h) have approximately the same boundaries under both FO and FO+FM control assumptions. However, an additional FM controlled PMA is identified in Figure 5h (red), which was not partitioned in the case considering only FO PMAs. This point illustrates that the strategy the utility has with respect to type of PMA control affects the outcome of how the network is most effectively partitioned.

Illustrating the order of magnitude of the pressure reduction results, the particular Pareto optimal solution in Figure 5h yielded a pressure reduction of 0 (unreduced), 7.7, 14.8 and 15.4 mH_2_O (0, 75, 145 and 151 kPa) for PMA no. 1 to 4, with PMAs no. 1–3 containing approximately 19 km pipe network each, and PMA no. 4 containing 13.6 km pipe network.

**Figure 6:**
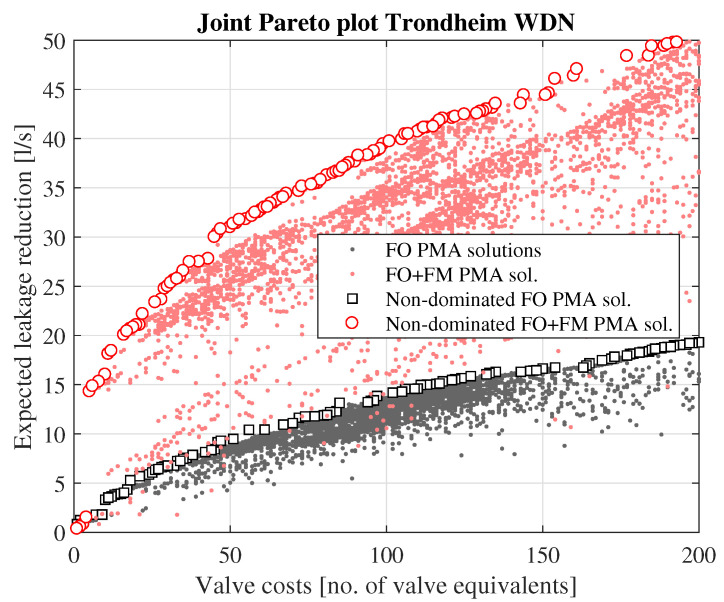
Resulting Pareto plot for the complete Trondheim WDN.

For the entire Trondheim WDN, the effectiveness of pressure reduction efforts is illustrated in Figure 6, which shows the Pareto plot for the whole system. The figure illustrates that dynamic pressure management is expected to be considerably more effective also on a system level. For instance, the expected leakage reduction using 20 valve equivalents is around 21.1 L/s if one uses both FO and FM PMAs, but only 4.8 L/s if one assumes FO-controlled PMAs only.

### 3.4. Cost Comparison and Economically Optimal Pressure Reduction Solution

To obtain an indication of the economic feasibility of the possible solutions, a simplified net-present value (NPV) analysis was performed for the candidate solutions, in which the total investment and maintenance cost of installing new PRVs was balanced against the economic value of the expected reduction in leakage losses (cost of water treatment and transport), and the NPV was maximised. Unit costs for valve purchase and maintenance, costs of water production, discount rates were estimated based on experiences from the Trondheim municipality (see Table 2). The total NPV was calculated as follows:

(5) $$\begin{array}{cc} C_{NPV}=\sum_{t=0}^{\infty }( & \frac{\Delta Q_{leak}\cdot c_{WL}}{{\left(1+\theta \right)}^{t}}-\frac{c_{mnt.FO}\cdot n_{FO}+c_{mnt.FM}\cdot n_{FM}}{{\left(1+\theta \right)}^{t}})-\sum_{t=0}^{\infty }\frac{c_{FO}\cdot n_{FO}+c_{FM}\cdot n_{FM}}{{\left(1+\theta \right)}^{t\cdot SL}} \end{array}$$

The NPV analysis was performed on every candidate solution of the Pareto front, after which the solution which maximised the NPV was chosen, i.e., the solution that yields the highest expected economic benefit for the Trondheim municipality.

For the case only considering FO PRVs, the economically most efficient solution identified is achieved by installing 24 FO PRVs, which yields a total expected NPV of 21 million NOK (Table 3). However, this solution only yields an expected reduction in the leakage loss of 1.3 percentage points, which is far less than Trondheim’s strategic goal (reduction from 28 to 20%).

Comparatively, the economically optimal solution, if one considers both FO and FM PRVs, yields a solution of a considerably larger magnitude, with 48 FM and 7 FO PRVs. Even though this solution entails substantially higher investment and maintenance costs, this is outweighed by a much more effective expected leakage loss reduction (6.0% point reduction). The total expected NPV of this solution is 106.5 million NOK, is approximately fivefold the expected NPV of the optimal solution considering only FO PRVs. The majority of the PRVs in the economically optimal solutions are placed on pipes in the diameter range 150–200 mm, with a few PRVs exceeding (typically 250–300 mm).

The resulting configuration for the 30 PMAs with the largest expected leakage reduction impact, identified in the economically optimal solution, are rendered in Table 4 (a map depicting the configurations of the resulting PMAs is not shown, as publishing a map representation of the entire WDN is in violation of Trondheim municipality’s data security protocols); the entries in the table are sorted in descending order by the expected leakage reduction impact in each newly created PMA. It is specified if the pressure reduction for the each PMA is to be static (FO PR) or dynamic (FM PR), and how many new PRVs need to be installed in order to create the PMA (0 indicates that no new PRV needs to be installed, and that the pressure reduction can be achieved by adjusting existing PRVs). These 30 zones make up approximately 95% of the expected leakage reduction loss in the economically optimal solution. In total, approximately 85% of the expected leakage reduction will occur in zones assigned for dynamic pressure management.

The results in Table 4 clearly demonstrate that dynamic (FM) pressure reduction will be instrumental in achieving effective leakage reduction in Trondheim, as the 17 most highly ranked zones identified for pressure reduction area assumed to be FM zones, and only seven of the 30 most highly ranked zones are assumed to be FO-controlled zones. The FM-controlled zones in the identified optimal solution range from large zones with moderate pressure reduction (e.g., zone no. 3, which contains 92.6 km of pipe and a maximum pressure reduction of only 4.9 mH_2_O (48 kPa)), to smaller zones with substantial pressure reduction (e.g., zone 10, with only 4.4 km of pipe and a maximum pressure reduction of 52.1 mH_2_O (511 kPa)). The zones identified for FO pressure reduction are generally smaller than those identified for FM pressure reduction, with the largest one containing 7.7 km of pipe network.

## 4. Discussion

This paper set out to develop, describe and test a method for identifying effective pressure reduction solutions for WDNs that are subject to critical capacity requirements. The overall objective of these efforts has been to facilitate the implementation of pressure management solutions in complex WDNs where capacity requirements are maintained through looped networks, and furthermore to assess to which extent pressure management can contribute to reduce leakage losses in Norwegian WDNs. To demonstrate the developed methods, a case study was performed using Trondheim’s WDN.

A simple economical analysis was performed on the identified Pareto optimal pressure reduction solutions. The economical analysis showed that pressure reduction can be an economically viable solution for reducing water loss in Trondheim’s WDN, both for the case assuming that one only uses FO pressure regulation, as well as for the case assuming a combination of FO and FM pressure regulation. However, the analysis also showed that the optimal solution using a combination of FO and FM PRVs is much more effective, both in terms of expected leakage reduction and NPV gain, compared to the optimal solution assuming only FO PRVs. This is mainly caused by the fire-fighting capacity requirements. Large parts of Trondheim’s WDN is grossly over-dimensioned with respect to normal day-to-day demand situations, with considerable excess pressure capacity that can be reduced, without compromising the service. However, for many areas of the WDN, this excess pressure capacity is necessary for ensuring positive pressures when extracting fire-fighting demands. If one uses only FO PRVs, one can only reduce the pressure to the fixed setting at which minimum pressures are ensured in the event of fire-fighting demand extraction in any of the network nodes. However, if one uses FM PRVs, one can reduce the pressure dynamically; during normal, diurnal demand variations the pressure can be reduced to the point at which minimum service pressure is ensured in all nodes, whereas in the event of a fire-fighting demand, the PRVs can be configured so that they reduce the pressure less, thus providing the capacity necessary for the fire-fighting demand, and at the same time ensuring minimum service pressure in all nodes. Although the investment and maintenance costs for FM PRVs are considerably higher compared to FO PRVs, the economic analysis shows that this is outweighed by the added leakage reduction benefit that can be achieved, due to this extra potential for pressure reduction entailed in the dynamic control. The difference in effectiveness between FO- and FM-based pressure management solutions is also supported by the Pareto plots (see, e.g., Figure 3, Figure 4, Figure 5 and Figure 6).

Trondheim’s strategic objective is to reduce their leakage loss from 28 to 20%, and using pressure reduction is assumed to be a pivotal component in achieving this objective. The results from the analyses in this paper demonstrate that pressure management can be an economically viable option for achieving some of this desired leakage reduction, provided that one chooses a strategy which entails the use of dynamic (FM) pressure control for a considerable number of the newly identified PMAs. With an economically optimal solution expected to reduce the leakage loss by 6% points, it is reasonable to assume that dynamic pressure management can contribute significantly to achieve the strategic objective set by the Trondheim municipality. Traditional, static (FO) pressure management solutions are expected to have considerably less leakage reduction impact. The finding that FM-based pressure management often is more economically effective compared to FO-based pressure management in WDNs with high flow variability concurs with other case studies in earlier research [12,38,39].

Several of the identified dynamically regulated PMAs will depend on multi-inlet supply, combined with RTC, to accommodate for changes in the demand situations. This can be achieved by utilising novel sensor and transmission technology. There exists a sufficient understanding of how to robustly and effectively control single-inlet valve systems under steady-state/gradual variations in demand [12]. However, there is a need to perform additional research on how to implement sensor and control for systems in multi-inlet PMAs subject to potentially abrupt changes in demand caused by, e.g., hydrant openings [12,40,41], and ensure robust, reliable flow and pressure conditions, as well as absence of valve hunting oscillations. This research should determine the need and location of pressure and flow sensors in the multi-inlet PMAs, and how the ensemble of PRVs regulating the pressure should be jointly controlled based on the real-time input from these sensors.

The method described in this paper is not constrained to generate only nested solutions, i.e., where solutions with a higher number of cuts are generated from solutions with a lower number of cuts [17]. This constraint was considered too restrictive for the partitioning of the PMAs, as there is no guarantee that the most effective exploitation of the available pressure reduction potentials is actually achieved through nested PMA partitioning solutions, especially in the case where both FO and FM PMAs are considered simultaneously. However, visual inspection of the results shows that the solutions in practiceconverge towards nested solutions in some cases (see, e.g., Figure 4). The identification of effective nested solutions can be relevant in cases where one plans a gradual or stepwise implementation of new PMAs, which may be an argument for including this constraint in the optimisation algorithm. However, the results from the Trondheim case study also demonstrate that the strategy one chooses with respect to the use of FO and FM control of the new PMAs will influence the optimal partitioning of the network. Generally, the use of PMAs with FM control favours larger partitions, typically in which the potential for dynamic pressure reduction is considerably higher than the static pressure reduction potential, whereas the use of PMAs with FO control tends to favour smaller, branched substructures of the network. The implementation of nested solutions, without a strategy with respect to FO/FM control is thus not advisable, as this strategy strongly will affect the network partitioning.

The actual effectiveness of the identified optimal solutions is contingent on the accuracy the parameters describing the leakage loss in the system [42], namely the background leakage coefficients and exponents. The leakage parameters for Trondheim’s WDN model have been calibrated based on data from the existing DMAs. However, the Trondheim municipality is currently effectuating a project in which they are increasing the number of DMAs in their WDN, by installing new water meters and sub-dividing existing DMAs into smaller areas. The data from the new DMAs will allow for a more refined calibration of the background leakage in the system, with better spatial resolution, and increased ability to distinguish leakage parameters for different cohorts of pipes. It will therefore be advisable to perform a recalibration of the leakage parameters, based on the new DMA metre data, to ensure more accurate input to the optimisation scheme described in this paper. The optimisation procedure could then be run again with the revised leakage parameters, thus ensuring more accurate and relevant recommendations, before finally concluding on the best strategy for PMA configuration in Trondheim.

## 5. Conclusions

This paper addresses the issue of how to identify effective solutions for reducing pressure, and consequently leakage losses, in complex, existing WDNs. A method for optimising system partitioning into PMAs, while at the same time conserving the required nodal fire-fighting capacity requirements, has been developed and demonstrated. Through a case study using Trondheim’s WDN, it has been demonstrated how this method could be used to support decision-making regarding effective management of pressures for leakage reduction in an existing system, distinguishing between using traditional static (FO) and dynamic (FM) regulation to manage the pressure in the identified PMAs. The results show that pressure management can be an economically effective way of achieving a considerable proportion of their leakage reduction goal, provided that the lion’s share of the investments in new PMAs are directed towards dynamically (FM) regulated PMAs.

The economical optimisation demonstrates that one can expect as much as 4–5 times more leakage reduction if one choses the optimal solution using a combination of FM and FO PRVs (expected leakage reduction of 43.27 L/s, or 6.0%-points), with 85% of the expected leakage reduction occurring in PMAs assigned for dynamic pressure control, compared to the economically optimal solution assuming only FO PRVs (expected leakage reduction of 9.08 L/s, or 1.3%-points). The difference in cost saving potential is on the same order of magnitude, with an expected NPV saving of 106.5 million NOK for the solution combining FO and FM PRVs, versus 21.2 million NOK for the FO PRVs only solution. The resulting Pareto plots also demonstrate that the combination of FO and FM PRVs often is expected to be 3–5 times more effective in terms of leakage reduction, compared to using only FO PRVs.

The optimised pressure reduction solution for Trondheim’s WDNs includes a combination of simple, smaller (<7.7 km) single-inlet FO-controlled PMAs, and FM-controlled PMAs of varying size (1.2–92.6 km) and pressure reduction potential (4.9–52.1 mH_2_O (48–511 kPa)). Some of the FM-controlled PMAs will rely on multiple inlets to maintain the fire-fighting requirements. Effective implementation of pressure reduction measures in the case study area will thus depend on further research to enable installation and management of robust, stable and reliable RTC-based solutions for controlling multi-inlet FM PMAs, which can react appropriately and timely to abrupt changes in water demand, such as fire hydrant activation, without causing hydraulic instability. If this can be achieved for Trondheim’s WDN, it is expected that Trondheim may reduce their leakage proportion from 28% to 22% by means of pressure management, which is close to their target of 20%.

## Figures and Tables

**Table 1:** Expected (Pareto) optimal leakage reduction achieved for alternative number of valve equivalents for example zone (no. 106).

Valve	Leakage Reduction [L/s]	
Equiv.	FO	FO+FM
1	0.064	0.064
2	0.011	0.320
3	0.136	0.382
4	0.158	0.572
5	0.178	0.591
6	0.196	0.626
7	0.216	0.646
8	0.234	0.708

**Table 2:** Input parameters used to calculate maximal NPV of pressure reduction measures.

Item		Value	Unit
Cost of water production & transport	$c_{WL}$	6.5	NOK */m^3^
Capital investment cost FO-PRV	$c_{FO}$	250,000	NOK
Capital investment cost FM-PRV	$c_{FM}$	500,000	NOK
Yearly maintenance cost FO-PRV	$c_{mnt.FO}$	15,000	NOK/year
Yearly maintenance cost FM-PRV	$c_{mnt.FM}$	30,000	NOK/year
PRV service life	SL	40	years
Discount rate	θ	5	%

* NOK refers to the currency Norwegian Kroner; 1 NOK equals approximately 0.1 Euros.

**Table 3:** NPV results for case considering only FO PMAs and considering combination of FO and FM PMAs (FO+FM PMA).

Strategy	Only FO PMAs	FO+FM PMAs	Unit
Exp. leakage loss reduction	9.08	43.27	L/s
Exp. leakage reduction	1.3	6.0	% points
Number of new FO-PRVs	24	7	-
Number of new FM-PRVs	-	48	-
Cost of new PRVs installed	12.5	53.6	mill. NOK
Cost of reduced water loss	33.7	160.1	mill. NOK
NPV of solution	21.2	106.5	mill. NOK

**Table 4:** Resulting configuration for the 30 most important PMAs in the economically optimal solution identified when considering both FO and FM pressure reduction, ordered by expected leakage reduction if realised.

	No. of	Pipe	FO PRV	FM PRV
	New	Length in	Reduction	Reduction
Zone	PRV	Zone [km]	[mH_2_O] *	[mH_2_O] *
1	1	23.8	-	26.6 (261)
2	2	13.0	-	38.5 (378)
3	2	92.6	-	4.9 (48)
4	2	20.1	-	21.1 (207)
5	1	38.6	-	10.9 (107)
6	3	24.7	-	14.0 (137)
7	2	38.6	-	7.9 (77)
8	2	13.2	-	22.3 (219)
9	1	26.5	-	8.8 (86)
10	3	4.4	-	52.1 (511)
11	2	13.4	-	15.6 (153)
12	3	13.6	-	15.4 (151)
13	1	5.9	-	33.0 (324)
14	1	16.9	-	10.5 (103)
15	2	4.2	-	37.7 (370)
16	2	3.8	-	34.8 (341)
17	2	3.6	-	35.9 (352)
18	1	2.9	42.7 (419)	-
19	1	17.1	-	7.1 (70)
20	2	2.1	-	55.7 (546)
21	1	26.3	-	4.1 (40)
22	1	6.1	-	17.5 (172)
23	1	3.2	-	22.7 (223)
24	1	1.8	40.3 (395)	-
25	0	7.7	8.1 (79)	-
26	0	6.3	8.6 (84)	-
27	1	3.3	15.0 (147)	-
28	0	2.1	22.8 (224)	-
29	1	1.2	-	38.8 (373)
30	0	2.7	15.9 (156)	-

* (kPa) in round brackets.

**Figure A2:**
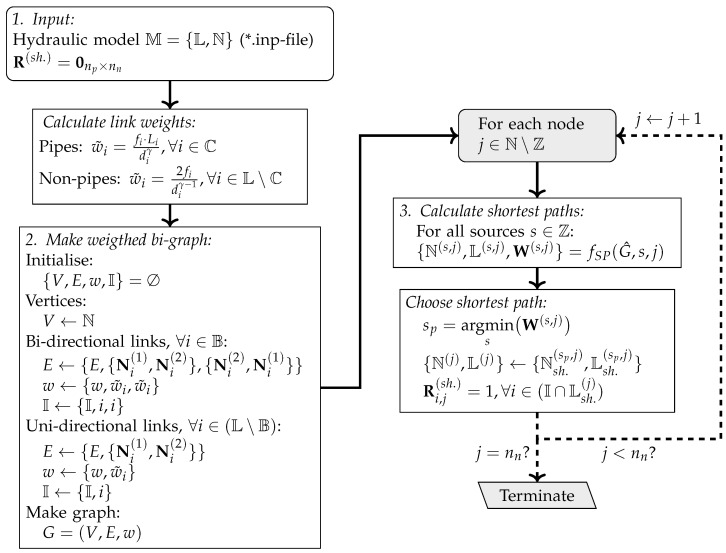
Shortest path identification algorithm.

## Data Availability

The data used to support the findings presented in this paper are not available for sharing, due to the fact that the data describe details about an actual water distribution system which is considered a critical infrastructure.

## Appendix A

### Appendix A.2

The algorithm for ensuring algebraic connectivity is depicted in Figure A2 and contains the following steps:

(1) A $n_{p}$ by $n_{n}$ matrix, $R^{\left(sh.\right)}$, is initialised. $R_{i,j}^{\left(sh.\right)}=1$ if link *i* is in the shortest supply path to *j*, and 0 otherwise.
(2) A graph *G* is made:
  (a) The set of nodes N in the hydraulic model is assigned as vertices *V* in *G*;
  (b) The set of links L are represented as weighted edges *E* in *G*;
  (c) Each link *i* is assigned a weight $w_{i}$ according to its hydraulic resistance, based on its length $L_{i}$, its diameter $d_{i}$, some measure of its friction loss $f_{i}$: $w_{i}=\frac{f_{i}L_{i}}{d_{i}^{\gamma }}$ for pipes (∀i∈C) and $w_{i}=\frac{2f_{i}}{d_{i}^{\gamma }}$ for-non pipe links (∀i∈L\C), according to the assumptions for friction loss calculation in [31];
  (d) Links that allow bi-directional flow (i∈B), like pipes, are represented twice in the graph (one edge for each flow direction), while links that allow only uni-directional flow (i∈L\B), e.g., active valves and pumps, are represented only in the allowed flow direction, thus avoiding that shortest paths can be identified in the opposite direction of these links’ flow directions (which could be the case if an undirected graph was used).
  Result: G=(V,E,w)
(3) For each junction *j* the shortest paths in supplying to this junction are identified in *G*:
  (a) The shortest path to junction *j* from any of the source nodes s∈Z⊂N are identified using Dijkstra’s algorithm [35] on the graph *G*;
  (b) The shortest of the paths from any of the nodes to junction *j* is chosen as the path which must be conserved in order to maintain algebraic connectivity to junction *j*, the links in this path are denoted $L^{\left(j\right)}$;
  (c) All links in $L_{sh.}^{\left(j\right)}$ are considered as necessary for maintaining the function in junction *j*; thus, junction *j* depend on all links in $L^{\left(j\right)}$: $R_{i,j}^{\left(sh.\right)}=1,\forall i\in \left(I\cap L_{sh.}^{\left(j\right)}\right)$.

## References

[1] Salas A.M., Biancalani R., Chocholata L. (2018). Progress on Level of Water Stress—Global Baseline for SDG Indicator 6.4.2.

[2] Statistics Norway (2019). Municipal Water Supply. https://www.ssb.no/natur-og-miljo/statistikker/vann_kostra.

[3] AL-Washali T., Sharma S., Lupoja R., AL-Nozaily F., Haidera M., Kennedy M. (2020). Assessment of water losses in distribution networks: Methods, applications, uncertainties, and implications in intermittent supply. Resour. Conserv. Recycl..

[4] Sanchez-Rodriguez R. (2011). Water and Wastewater.

[5] Mattilsynet (2014). Nasjonale Mål—Vann og Helse Vedtatt av Regjeringen 22 Mai 2014.

[6] Krogh A., Grindheim T., Hem L., Wermskog L., Kjøglum K.T., Olsen M., Hult F., Høysæter T. (2015). Hovedplan Vannforsyning 2015–2030.

[7] Kommune B. (2015). Hovedplan for Vannforsyning 2015–2024.

[8] Misund A.K., Johnsen K.G., Kierulf H., Holen E., Tveit O.A., Berg R., Lynum Ø., Bollingmo Å. (2017). Hovedplan for Vannforsyning, Vannmiljø og Avløp 2011–2022.

[9] Østensjø I., Skjørestad L., Jacobsen B.Z. (2010). Hovedplan for Vannforsyning, Vannmiljø og Avløp 2011–2022.

[10] Ulanicki B., AbdelMeguid H., Bounds P., Patel R. (2009). Pressure Control in District Metering Areas with Boundary and Internal Pressure Reducing Valves. Water Distribution Systems Analysis 2008.

[11] Awad H., Kapelan Z., Savić D. (2009). Analysis of Pressure Management Economics in Water Distribution Systems. Water Distribution Systems Analysis 2008.

[12] Vicente D., Garrote L., Sánchez R., Santillán D. (2016). Pressure Management in Water Distribution Systems: Current Status, Proposals, and Future Trends. J. Water Resour. Plan. Manag..

[13] Haider H., Al-Salamah Ibrahim S., Ghazaw Yousry M., Abdel-Maguid Ramadan H., Shafiquzzaman M., Ghumman Abdul R. (2019). Framework to Establish Economic Level of Leakage for Intermittent Water Supplies in Arid Environments. J. Water Resour. Plan. Manag..

[14] Piller O., van Zyl J.E. (2014). Incorporating the FAVAD Leakage Equation into Water Distribution System Analysis. Procedia Eng..

[15] Schwaller J., van Zyl J. (2014). Modeling the Pressure-Leakage Response of Water Distribution Systems Based on Individual Leak Behavior. J. Hydraul. Eng..

[16] Schwaller J., van Zyl J.E. (2014). Implications of the Known Pressure-response of Individual Leaks for Whole Distribution Systems. Procedia Eng..

[17] Laucelli D.B., Simone A., Berardi L., Giustolisi O. (2017). Optimal Design of District Metering Areas for the Reduction of Leakages. J. Water Resour. Plan. Manag..

[18] Zhang Q., Wu Z.Y., Zhao M., Qi J., Huang Y., Zhao H. (2016). Leakage Zone Identification in Large-Scale Water Distribution Systems Using Multiclass Support Vector Machines. J. Water Resour. Plan. Manag..

[19] di Nardo A., Di Natale M., Santonastaso G.F., Venticinque S. (2013). An Automated Tool for Smart Water Network Partitioning. Water Resour. Manag..

[20] di Nardo A., di Natale M., Santonastaso G.F., Tzatchkov V.G., Alcocer-Yamanaka V.H. (2014). Water Network Sectorization Based on Graph Theory and Energy Performance Indices. J. Water Resour. Plan. Manag..

[21] Alvisi S. (2015). A New Procedure for Optimal Design of District Metered Areas Based on the Multilevel Balancing and Refinement Algorithm. Water Resour. Manag..

[22] Brentan B., Campbell E., Goulart T., Manzi D., Meirelles G., Herrera M., Izquierdo J., Luvizotto E. (2018). Social Network Community Detection and Hybrid Optimization for Dividing Water Supply into District Metered Areas. J. Water Resour. Plan. Manag..

[23] Zhang Q., Wu Y.Z., Zhao M., Qi J., Huang Y., Zhao H. (2017). Automatic Partitioning of Water Distribution Networks Using Multiscale Community Detection and Multiobjective Optimization. J. Water Resour. Plan. Manag..

[24] Gutiérrez-Pérez J., Herrera M., Pérez-García R., Ramos-Martínez E. (2013). Application of graph-spectral methods in the vulnerability assessment of water supply networks. Math. Comput. Model..

[25] Liu J., Han R. (2018). Spectral Clustering and Multicriteria Decision for Design of District Metered Areas. J. Water Resour. Plan. Manag..

[26] Perelman L., Ostfeld A. (2011). Topological clustering for water distribution systems analysis. Environ. Model. Softw..

[27] Diao K., Fu G., Farmani R., Guidolin M., Butler D. (2016). Twin-Hierarchy Decomposition for Optimal Design of Water Distribution Systems. J. Water Resour. Plan. Manag..

[28] Ciaponi C., Murari E., Todeschini S. (2016). Modularity-Based Procedure for Partitioning Water Distribution Systems into Independent Districts. Water Resour. Manag..

[29] Rokstad M.M. (2021). Quick verification of hydraulic capacity requirement compliance under segmentation of looped networks.

[30] DIBK (2017). Veiledning om Tekniske Krav Byggverk—Direktoratet for Byggkvalitet.

[31] Rossman L. (2000). Epanet 2.0 User’s Manual. Water Supply and Water Resources Division.

[32] Giustolisi O., Savic D., Kapelan Z. (2008). Pressure-Driven Demand and Leakage Simulation for Water Distribution Networks. J. Hydraul. Eng..

[33] Muranho J., Ferreira A., Sousa J., Gomes A., Marques A.S. (2014). Pressure-dependent Demand and Leakage Modelling with an EPANET Extension—WaterNetGen. Procedia Eng..

[34] Statistics Norway (2020). Population and Land Area in Urban Settlements. https://www.ssb.no/en/befolkning/folketall/statistikk/tettsteders-befolkning-og-areal.

[35] Dijkstra E.W. (1959). A note on two problems in connexion with graphs. Numer. Math..

[36] Deb K., Pratap A., Agarwal S., Meyarivan T. (2002). A fast and elitist multiobjective genetic algorithm: NSGA-II. IEEE Trans. Evol. Comput..

[37] Lambert A., Hirner W. (2000). Losses from Water Supply Systems: A Standard Terminology and Recommended Performance Measures.

[38] Creaco E., Walski T. (2017). Economic Analysis of Pressure Control for Leakage and Pipe Burst Reduction. J. Water Resour. Plan. Manag..

[39] Kunkel G., Sturm R. (2011). Piloting proactive, advanced leakage management technologies. J. AWWA.

[40] Creaco E., Campisano A., Modica C. (2018). Testing behavior and effects of PRVs and RTC valves during hydrant activation scenarios. Urban Water J..

[41] Campisano A., Modica C., Reitano S., Ugarelli R., Bagherian S. (2016). Field-Oriented Methodology for Real-Time Pressure Control to Reduce Leakage in Water Distribution Networks. J. Water Resour. Plan. Manag..

[42] Rokstad M.M., Ugarelli R.M. (2017). Investigation of the Ability to Accurately Estimate Background Leakage Parameters in WDS Network Simulation Models. J. Water Resour. Plan. Manag..

